# Interactions between ENDS and cigarette use: evidence from a 2022 national telephone survey in South Africa

**DOI:** 10.1136/tc-2023-058521

**Published:** 2024-05-24

**Authors:** Kirsten van der Zee, Corné Van Walbeek

**Affiliations:** 1 Research Unit on the Economics of Excisable Products, School of Economics, University of Cape Town, Rondebosch, South Africa

**Keywords:** Electronic nicotine delivery devices, Cessation, Low/Middle income country, Nicotine

## Abstract

**Introduction:**

Electronic nicotine delivery systems (ENDS) may serve as a cessation tool for people who smoke cigarettes. However, for people who do not smoke, ENDS may be a gateway to nicotine addiction and cigarette use. This paper aims to quantify these behaviours in South Africa.

**Methods:**

We analysed a nationally representative telephone survey of 21 263 South Africans living in urban areas. For those respondents who had used both products (N=771), we developed a typology that describes the sequence in which cigarette and ENDS initiation occurred. ‘On-rampers’ describe people who used ENDS first and later initiated cigarette smoking. ‘Off-rampers’ describe people who used cigarettes first, took up ENDS and later quit cigarettes while still using ENDS. ‘Failed off-rampers’ describe people who started using ENDS while smoking cigarettes but later quit using ENDS. ‘Continuing dual consumers’ describe people still using both products at the time of the interview.

**Results:**

Of the overall sample (N=21 263), 1.7% used or had used ENDS but had no history of using cigarettes. Of dual consumers (N=771), 8.8% were classified as ‘on-rampers’, 13.9% as ‘off-rampers’, 20.9% as ‘failed off-rampers’ and 56.4% as ‘continuing dual consumers’. Roughly half of those classified as off-rampers, failed off-rampers or continuing dual consumers stated that they started using ENDS to help them quit cigarettes.

**Conclusions:**

The typology reveals a multifaceted relationship between ENDS and cigarette use in South Africa. Policy interventions should aim to minimise on-ramping and maximise off-ramping. Given the high prevalence of continued dual use and failed off-ramping, targeted cessation support should be provided for people who use ENDS and are trying to quit cigarettes.

**WHAT IS ALREADY KNOWN ON THIS TOPIC**

- Broadly, there are two schools of thought regarding electronic nicotine delivery systems (ENDSs): (1) ENDSs are a cessation tool for cigarette smoking and (2) ENDSs are inherently harmful, and, more importantly, they lead people who do not use tobacco to become addicted to nicotine, and potentially to start smoking cigarettes. The research regarding ENDS as a cessation tool is inconclusive, with some studies finding a strong positive effect of ENDS on smoking cessation, and others finding that ENDS have no, or even a negative, effect on quitting cigarettes. Research shows that ENDS use among people who do not smoke, particularly young people, is rapidly increasing. A limited number of studies have investigated these effects in South Africa.

**WHAT THIS STUDY ADDS**

- In this paper, we created a sequence-of-use typology for ENDS and cigarettes. The study found both ‘on-ramping’ and ‘off-ramping’ behaviour in South Africa. This suggests that ENDSs have played a role in both cigarette smoking initiation and cigarette smoking cessation.

**HOW THIS STUDY MIGHT AFFECT RESEARCH, PRACTICE OR POLICY**

- Policy-makers should carefully weigh the potential harms and benefits of ENDS use when formulating regulations around ENDS. They should also aim to minimise ENDS use among youth and people with no history of smoking but should not impose unnecessary impediments to their use for those who want to quit smoking.

## Introduction

 Since their invention, electronic nicotine delivery systems (ENDSs) have gained popularity globally. A 2022 systematic review, which covered four continents, found that the prevalence of regular ENDS use in those continents was around 11% (North America 10%, Asia 14%, Europe 11% and Australia/Oceania 6%).^1^ ENDS use in Africa was not considered in this study, but it seems likely to be substantially lower than in the rest of the world.^2^

There has been much debate about where ENDS should fall in the broader tobacco-control conversation. The tobacco industry and ENDS proponents frame ENDS in the context of ‘harm reduction’.^3^ They view ENDS as a potentially life-saving alternative for people who smoke cigarettes because ENDS can deliver nicotine with fewer toxicants than cigarettes.^4^ In this view, vaping is a smoking-cessation tool and is marketed primarily to people who smoke cigarettes.

Many public health officials and advocates, on the other hand, are concerned about the potential harms of ENDS. One consideration is that vaping itself presents health risks.^5^ In addition to this, there is a concern that vaping has the potential to serve as a gateway to cigarette smoking, by addicting people to nicotine.^6^ Young people, in particular, might be attracted to ENDS because of their novel product design and enticing flavours. Sceptics also emphasise that ENDSs have been used to deliver other drugs, such as tetrahydrocannabinol (the psychoactive component of marijuana), methamphetamine and others.^7^

There is grave concern in the South African tobacco-control community about rising rates of ENDS use, particularly among young people.^8^ A recent non-representative survey of South African high schools indicated that 15.3% of school students had used a vape device in the previous 30 days; this was much higher than the 4% who had smoked tobacco in the previous 30 days.^9^ The concern about ENDS use in South Africa is exacerbated because a recent study using online survey data found that e-cigarette use depressed long-term cigarette cessation in the sample.^10^

Currently, the only regulation on ENDS products is an excise tax on e-liquids, which was imposed in June 2023. The government is currently in the process of passing legislation that will regulate ENDS as tobacco products. However, this has been a drawn-out process, with push-back from the tobacco and vaping industries.^11^

The aim of this study is to contribute to the literature that balances the potential harms and benefits of ENDS use.^4 12^ To do this, we analyse a nationally representative survey of adult South Africans living in urban areas. The survey questions focus on ENDS and cigarette use. We use the sequence of initiation and cessation of ENDS and cigarettes to assess how the two products are used in relation to one another. This provides insight into which people might have benefited from ENDS use, and for which people ENDS use might have introduced unnecessary harm. In addition, we assess respondents’ reported reasons for initiating ENDS use, to validate whether these align with what might have been expected. For example, did the adults who smoked initiate vaping with the intention of quitting cigarettes, or as a complement to cigarette smoking? While ENDS use among under 18s is important to this debate, under 18s were not surveyed because of the nature of the data collection ethics approval.

## Methods

This paper is based on the South African E-cigarette Survey 2022 (SAES), a telephone survey conducted between January 2022 and September 2022.^13^ The survey was designed to be representative of adults (18 years and older) living in urban South Africa.

The survey was designed with a target quota of 1250 completed interviews by people who used ENDS at the time of the interview or had done so in the past. There was also a quota of 700 completed interviews by people who smoked traditional/manufactured cigarettes (‘cigarettes’) at the time of the interview or had done so in the past. To fill these quotas, 21 263 adults in total were interviewed. Respondents answered a questionnaire about their use of ENDS and cigarettes. Most respondents (16 658) indicated that they did not use ENDS or cigarettes.

The data collection followed a stratified random sample approach to achieve a representative sample. The stratification variables included province, geographical type (urban metro and urban non-metro), population/race group and a neighbourhood income index. Because of the large differences in population sizes of the strata, the sample was disproportionately drawn from smaller strata to ensure that they were adequately represented. Survey weights are applied throughout the analysis to account for this disproportionate sampling. Details about the stratification and weighting are outlined in the SAES user manual.^14^

The survey collected information on respondents’ past and present use of both cigarettes and ENDS. Regular ENDS use refers to at least weekly use (in the past or at the time of the interview). Regular cigarette use refers to people who have smoked at least 100 cigarettes in their lifetime and smoke/smoked at least weekly (in the past or at the time of the interview). People who experimented with cigarettes or ENDS, but never used them regularly, are coded as if they never used that product.

Respondents who regularly used (at the time of the interview) or had regularly used, both cigarettes and ENDS simultaneously at some time are classified as ‘dual consumers’. For this group, we were interested in the sequence of use for cigarettes and ENDS. Respondents were asked at what age they started using each product and, for people who had quit, how long ago they quit. Respondents were also asked which product they started using first.

The dataset includes 1268 respondents who used ENDS, 901 of whom had also used cigarettes at some point. Of these, 46 were not included in the analysis because of incomplete information (eg, they did not report the year they started using ENDS). A further 32 respondents were excluded from the analysis because they reported starting using ENDS before 2003, which is when ENDS (as we know them) came onto the market. Of the remaining 823 respondents, 777 had regularly used cigarettes and ENDS simultaneously at some point (‘dual consumption’) and are placed into the sequence-of-use typology. In addition, the data include 367 respondents who only used ENDS (who did not and had never smoked cigarettes regularly), whom we also include in some parts of the analysis.

Each dual consumer was categorised into the sequence-of-use typology for ENDS and cigarette use.Table 1 describes the typology, with a visual representation of the sequence for each category. The dual consumers in categories A1−A3 and F started vaping before smoking cigarettes, dual consumers in category B started using both products in the same year and dual consumers in categories C1−E and G started smoking cigarettes before vaping.

**Table 1:** Typology of cigarette and ENDS sequence of use

	T0	T1	T2	Tsurvey	Description	Use type	Freq	Weighted share
A1					Started ENDS first, then later: > Took up cigarettes, still use both.	On-ramp	40	6.4
				­				
A2					> Took up cigarettes, quit ENDS, and still use cigarettes.	On-ramp	8	1.0
				­				
A3					> Took up cigarettes, and ultimately quit both (varying orders of quitting).	On-ramp	4	0.6
­								
B					Started smoking cigarettes and ENDS in the same year; with various outcomes: > Still use both. >Quit one product, still use the other. >Quit cigarette smoking and ENDS in the same year.	Potential on-ramp	7	0.7
­								
­								
­								
C1					First smoked cigarettes, then later: > Took up ENDS. Then quit cigarettes; still use ENDS.	Off-ramp	30	3.4
­								
C2					> Took up ENDS and quit cigarettes in the same year; still use ENDS.	Off-ramp	42	5.0
­								
C3					> Took up ENDS. Then quit cigarettes; later quit ENDS.	Off-ramp	9	1.6
­								
C4					> Took up ENDS. Quit ENDS and cigarettes in the same year.	Off-ramp	25	2.9
­								
D					First smoked cigarettes, then took up ENDS. Currently use cigarettes and have quit ENDS.	Failed off-ramp	160	20.7
­								
E					First smoked cigarettes, then took up ENDS. Currently use ENDS and cigarettes	Continuing dual use	440	55.9
­								
F					Started ENDS first, temporarily took up cigarettes, still use ENDS.	On-ramp; off-ramp	6	0.8
­								
G					First smoked cigarettes, then temporarily took up ENDS and quit ENDS; later quit cigarettes.	N/A	6	0.8
­								
Total					All dual consumers		777	100

T_0_ – T_2_ indicate time periods before the interview, and T_survey_ represents the time of the interview. Pink bars illustrate the cigarette use period, and orange bars illustrate the ENDS use period.

ENDS, electronic nicotine delivery system.

The ‘use type’ column provides a classification for each use sequence. In this classification, we use the terms ‘on-ramp’ and ‘off-ramp’. On-rampers are defined as dual consumers whose sequence of use suggests that ENDS use might have led to cigarette use. In other words, they used ENDS without ever having used cigarettes regularly, and later went on to start smoking cigarettes. In our typology, respondents categories A1−A3 and F are regarded as on-rampers because they initiated ENDS use with no reported history of regular cigarette use, and then started smoking cigarettes after using ENDS.

Category B respondents reported starting using ENDS and cigarettes in the same year. It is possible that in the absence of ENDS, these people would not have started smoking cigarettes. However, the opposite may also be true; in the absence of cigarette initiation, they may not have started using ENDS. For this reason, we term this group ‘potential on-rampers’.

Off-rampers, on the other hand, are those dual consumers whose sequence of use indicates that they quit cigarette smoking while using ENDS. Whether the person subsequently quit ENDS or not is irrelevant for the categorisation. In our typology, respondents in categories C1−C4 and F are considered off-rampers since they first smoked cigarettes, then took up ENDS, and subsequently, quit cigarettes while still using ENDS (note that category F exhibits features of both on-ramping and off-ramping so that this group is included in both definitions).

We regard category D as ‘failed off-rampers’ as these people temporarily took up ENDS while smoking cigarettes (and would have been classified as off-rampers if they had quit smoking cigarettes), but quit ENDS and continued smoking cigarettes. This group may have tried to use ENDS as a cessation tool but gave up. However, included in this group are people who may not have intended to quit smoking cigarettes with the use of ENDS.

Category E is classified as ‘continuing dual consumers’—this group initially smoked cigarettes, later took up ENDS and still used both products at the time of the interview. Some of these people may have taken up ENDS with the intention of off-ramping (quitting cigarettes) but had not done so by the time of the survey. It is also possible that these people had no intention of quitting cigarettes, but rather took up ENDS as a complement to cigarettes.

Category G does not fit the typology of on-ramping or off-ramping and is therefore not included in the results section; overall, 771 dual consumers are included in the results section below.

Lastly, survey respondents were asked ‘Why did you initially start vaping?’. This was an open-ended question, and the interviewer had a list of prepopulated responses (eg, ‘To help me stop smoking cigarettes’ or ‘It comes in flavours I like’) and could select as many options as applied. The interviewer also had the option to record a text response. In the data cleaning phase, this text response was either added to the prepopulated options (if appropriate) or was included in an ‘other’ category.

In the final part of the results section, the reasons for starting ENDS are summarised for each of the main use types (on-rampers, off-rampers, failed off-rampers and continuing dual consumers). We have included this in the results section because the concept of on-ramping and off-ramping makes implicit assumptions about the intentions of the different types of use. For example, one might assume that an off-ramper started using ENDS to help them quit smoking cigarettes, or that an on-ramper started vaping because they found the product or flavours enticing. By assessing the reasons quoted within each group, we get an indication of how well-aligned our expectations with respect to the revealed typology of use are to the reasons stated by the respondents.

## Results

### Data description

Of the total weighted sample, 1.7% had used ENDS without having ever used cigarettes, and 3.8% had used both ENDS and cigarettes (dual consumers).

**Figure 1:**
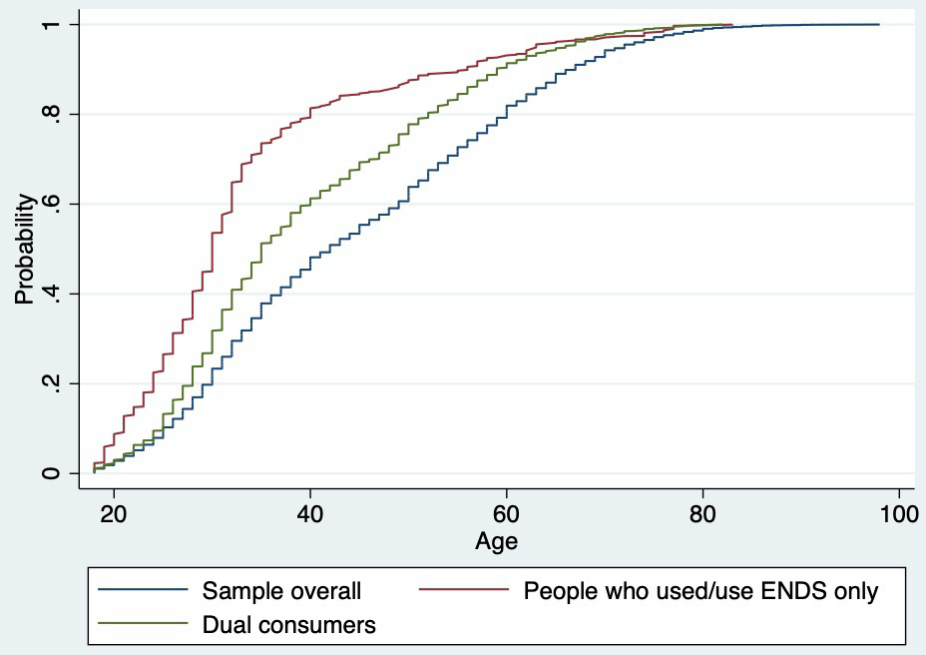
Cumulative distribution functions, by various group. We include three CDFs: (1) all respondents (including people who have never used ENDS and cigarettes), (2) people who used/use ENDS only and (3) dual consumers. CDFs, cumulative distribution functions; ENDS, electronic nicotine delivery system.

Figure 1 shows the cumulative distribution functions (CDFs) based on age. We include three CDFs: (1) all respondents (including people who have never used ENDS and cigarettes), (2) people who used/use ENDS only and (3) dual consumers. People who used/use ENDS only are the youngest group, with roughly 50% under the age of 30 and roughly 80% under the age of 40.

### Sequence of use

Table 2 places dual consumers into their respective sequence-of-use categories. Respondents who fall into categories that are uninteresting, based on the sequence-of-use typology (indicated by ‘N/A’ in table 1), are not included in this assessment.

**Table 2:** Use type description and duration of use and age analysis

Use type	Weighted share	Mean duration of use (in years)					Mean	Mean (10th; 90th percentiles)	Mean (10th; 90th percentiles)
		Use before dual	Dual use	Use postquitting	Total cigarette use	Total ENDS use	Current age	Age started cigarettes	Age started ENDS
On-rampers	8.0	1.8*	4.6	N/A	4.5	5.9	27.0	22.8 (17; 30)	20.7 (16; 28)
Potential on-rampers	0.7	0	4.6	N/A	5.2	4.9	28.8	21.8 (18; 33)	21.8 (18; 33)
Off-rampers	13.1	14.9†	1.4	2.3*	16.2	3.5	38.7	19.6 (16; 26)	34.5 (20; 54)
Failed off-rampers	20.9	18.0†	2.6	2.3†	22.5	2.6	41.7	19.2 (14; 26)	37.2 (21; 55)
Cont. dual consumers	56.4	14.9[Table-fn T2_FN2]	3.8	N/A	19.5	3.8	39.2	19.7 (15; 25)	33.8 (19; 53)
On-rampers and off-rampers	0.8	3.3*	6.5	0.9*	6.5	10.1	37.8	30.9 (19; 55)	27.7 (16; 53)
Total	100.0	14.4	3.3	2.2	18.4	3.8	38.6	19.9 (15; 26)	33.4 (18; 54)

* Denotes an ENDS use duration.

† Denotes a cigarette use duration.

ENDS, electronic nicotine delivery system.

#### On-ramping

On-rampers (including the 0.8% of people classified as dual consumers who also became off-rampers) made up 8.8% of the dual consumers. These respondents vaped for 1.9 years on average before taking up cigarettes and then smoked and vaped for an average of 4.7 years. Some of these people continued to use both cigarettes and ENDS at the time of the interview or had quit one product or the other, or both (see category A in table 1).

A very small percentage of dual consumers (0.7%) reported starting cigarettes and ENDS in the same year. We term these people ‘potential on-rampers’ because it is possible that, in the absence of ENDS, these people would not have started smoking cigarettes. If we assume that potential on-rampers initiated cigarette use because of their exposure to ENDS, then the share of on-rampers increases to 9.5% of dual consumers.

#### Off-ramping

Off-rampers made up 13.9% of dual consumers (this includes people who are classified as off-rampers and on-rampers, category F in table 1). Of this group, 70% were still using ENDS at the time of the survey. Off-rampers had smoked cigarettes for an average of 14.3 years before taking up ENDS, then dual-consumed for 1.8 years and continued vaping for an average of 2.2 years after quitting smoking.

Failed-off rampers made up 20.9% of dual consumers. These are people who smoked cigarettes, then started vaping, but quit ENDS and still smoked cigarettes at the time of the interview. These people are similar to the off-ramp group in that they smoked cigarettes for a long time before vaping (averaging 18 years prior to ENDS use). On average, they quit vaping after 2.6 years of dual use and continued to smoke cigarettes for another 2.3 years.

Continuing dual consumers are people who first smoked cigarettes and later took up ENDS. These respondents were still using both products at the time of the survey so could potentially still off-ramp. Continuing dual consumers made up the largest share of the dual consumer category (56.4%). These people smoked cigarettes for an average of 14.9 years before initiating ENDS.

Successful off-rampers quit cigarettes on average 1.4 years after taking up ENDS. Continuing dual consumers had been using both products for an average of 3.8 years (by the time of the interview), which is substantially longer than the dual-use period of successful off-rampers. Failed off-rampers used both products for an average of 2.6 years before quitting ENDS.

The averages in the text include the ‘on-ramper and off-ramper’ category (ie, category F in table 1), hence the deviation from the numbers reported in tables1  2 .

According to table 2, the average age of dual consumers was 38.6 at the time of the interview. On-rampers were younger (27.0 years) than off-rampers (38.7 years) (p=0.0), failed off-rampers (41.7 years) (p=0.0) and continuing dual consumers (39.2 years) (p=0.0). On-rampers also started smoking cigarettes at a significantly higher age (22.8 years) than other dual consumers did (<20 years) (p=0.0). On-rampers started using ENDS at 20.7 years old, which is much younger than other dual consumers (34.5 years) (p=0.0).

### Reasons for taking up ENDS

Table 3 reports the reasons that dual consumers provided for starting vaping. Note that respondents gave open-ended responses and interviewers could select more than one reason that fit the response (85% of respondents had one reason for starting, 12% had two reasons and 3% had three reasons or more).

**Table 3:** Reasons for starting vaping

	On-ramp	Off-ramp	Failed off-ramp	Continuing dual consumers	Total
Help quit/avoid cigarettes or other tobacco	12% (8)	58% (70)	49% (95)	37% (184)	40% (357)
Cool/new/curious/flavours	26% (18)	20% (25)	21% (41)	33% (159)	28% (243)
Family/friends vape	10% (8)	5% (7)	5% (12)	7% (39)	7% (66)
COVID-19 tobacco sales ban	0% (0)	3% (2)	7% (16)	3% (18)	4% (36)
Less harmful than cigarettes	0% (1)	1% (3)	6% (12)	2% (15)	3% (31)
More affordable than cigarettes	4% (1)	2% (2)	5% (10)	3% (13)	3% (26)
Stress	12% (7)	1% (1)	0% (0)	2% (11)	2% (19)
Use when cigarettes are not allowed	4% (2)	1% (1)	2% (3)	1% (9)	2% (15)
Other	32% (16)	8% (7)	5% (11)	11% (53)	11% (87)
Total	100% (61)	100% (118)	100% (200)	100% (501)	100% (880)

‘Potential on-ramp’ and ‘on-ramp; off-ramp’ are not reported due to the small sample size. The question was formatted for open-ended responses, and the interviewer could select more than one reason, if appropriate. The percentages refer to the weighted share by use type. The numbers in parentheses refer to the number of observations in the sample.

The main reason that on-rampers started using ENDS was that the products looked cool or new and because of the flavours (26%). 12% started vaping because of stress. A further 12% started using ENDS to avoid using cigarettes or other tobacco products. 10% started because their family or friends vaped. 32% of on-rampers reported reasons that did not fit into the main categories (‘other’).

More than half of off-rampers (58%) indicated that they started vaping to quit smoking cigarettes or other tobacco products. Among failed off-rampers, this figure was 49%, and among continuing dual consumers it was 37%. A substantial percentage of off-rampers (20%), failed off-rampers (21%) and continuing dual consumers (33%) indicated that they started vaping because the product looked cool or new, or they were attracted by the flavours. Other reasons for starting vaping are relatively minor in comparison with these two main reasons.

## Discussion

A major point of contention for public health officials in the area of tobacco control is how cigarettes and ENDS are used in relation to each other. Tobacco companies and ENDS proponents argue that ENDS are predominantly used by people who smoke cigarettes as a quitting aid because they deliver nicotine in a less harmful way. Studies have compared the effectiveness of ENDS as a cessation tool to that of nicotine replacement therapy (NRT).^12^,^26^ A number of studies (including randomised controlled trials, reviews, meta-analyses and longitudinal studies) have shown that ENDS outperform NRTs for cessation.^15^,^21^ However, the results are ultimately inconclusive since other studies have found no significant effect of ENDS on cigarette cessation,^22^,^27^ and others still (including longitudinal studies and meta-analyses) have found that ENDS use is associated with a significant reduction in cigarette cessation.^10 28 29^

For those who are able to quit smoking with the help of vaping, ENDS has the potential to reduce the health harms caused by tobacco use.^4^ In our dataset, off-rampers made up 13.9% of dual consumers. Of the dual consumers who might have used ENDS to quit cigarettes (ie, off-rampers, failed off-rampers and continuing dual consumers), 15% off-ramped. This is a crude estimate of the effectiveness of ENDS as a cessation tool in the sample. However, it probably underestimates ENDS’ cessation effectiveness because many of these people who smoked were not actually trying to quit cigarettes—only roughly 50% of this group said that they started using ENDS because they wanted to stop using cigarettes. Of the respondents who indicated that they wanted to use ENDS to help them quit cigarettes, 20% had off-ramped, 28% failed to off-ramp and 52% were still using both products at the time of the survey. 6% of off-rampers reported also using NRTs to help them quit; for these off-rampers, the use of NRTs confounds the effect of ENDS in cigarette-quitting success.

The duration analysis indicated that should a person who smokes cigarettes want to quit while vaping, they should do so fairly quickly; successful off-rampers quit cigarettes on average 1.4 years after starting ENDS while failed off-rampers and continuing dual consumers used both products for a longer period (2.6 and 3.8 years on average, respectively).

A unique feature of ENDS, compared with other cessation aids, is that they themselves pose risks, for example, an increased likelihood of lung and heart disease.^30 31^ While their health risks may be less than those of cigarettes, they are certainly not zero.^4 5^ For people who do not smoke cigarettes, taking up ENDS introduces its own harms^4 5^ and could be a gateway to tobacco use.^32^ Since the introduction of ENDS in 2003, many countries have experienced a significant rise in the use of these products, particularly among young people who have no history of cigarette smoking.^1 33^ These people are exposed to toxic substances and introduced to the risk of diseases and nicotine dependence.^34^ Our data revealed that 1.7% of urban-dwelling South African adults had used ENDS regularly, but had no history of cigarette smoking; these represent almost half a million adults. 70% of people with no history of cigarette smoking who used or use ENDS were below the age of 35.

Further to this, studies from a variety of countries have found a positive relationship between ENDS use and the subsequent initiation of cigarette smoking.^35^,^38^ Many tobacco control and public health advocates are concerned that ENDS are marketed to people who do not smoke, particularly to youth, and become a gateway to tobacco smoking.^39^ Proponents of ENDS tend to downplay the possibility that ENDS could be a gateway to nicotine addiction and subsequent cigarette use.^39^

Of the dual consumers who had no history of cigarette smoking, 8.8% displayed on-ramping behaviour. On-rampers made up 15% of all people who vaped with no history of cigarette use (this includes on-rampers—categories A and F in table 1—divided by the sum of on-rampers and people who only used ENDS, who had no prior history of cigarette use). The reasons these respondents reported for starting vaping were that they perceived the products as ‘cool’, they were curious about them, and they were attracted by the flavours. While the tobacco industry claims that ENDS are primarily marketed to people who smoke cigarettes, their marketing strategies clearly appeal to a new market segment, specifically those who have never smoked cigarettes. This is illustrated in figure 1—where the age distribution of people who only used ENDS was well below that of dual consumers and of the urban adult population overall—which indicates that people who use ENDS with no history of cigarette smoking are young. Among dual consumers, the on-rampers in the survey started vaping young (20.7 years old) compared with other dual consumers and started smoking cigarettes at a higher age than other people who smoked cigarettes. In the absence of ENDS, these respondents might never have started cigarette smoking.

There are a number of study limitations to consider. The data reflect respondents’ perceptions regarding the timing of initiation and quitting of each product. Incorrect recollections will result in measurement error. For example, a respondent might incorrectly state their age or the age at which they started using a product. In our case, recall error is likely to be random, and would thus cause a widening of the confidence intervals but would not bias the parameter estimates (proportions and means). Under 18s were excluded from the study because of feasibility issues (in South Africa, there are significant hurdles for surveys which include under 18s), and the data collection approach.

In this study, we have intentionally avoided directly comparing the number or share of on-rampers to off-rampers and making conclusions and policy recommendations based on such a comparison. Doing so would venture into discussions of bioethics, as this would require comparing the value of the lives potentially saved by ENDS as a cigarette cessation tool and the lives potentially lost as a result of ENDS use. There is currently a debate among researchers in the bioethics discipline regarding how to make these comparisons.^40 41^ The analysis in this paper is limited to measuring the various ENDS/cigarette use types and does not place a value judgement on which is more important.

Overall, our data show evidence of both on-ramping and off-ramping, and policy interventions should take cognizance of this. Presumably, policy-makers would want to maximise the off-ramp effect while minimising the on-ramp effect. This implies a nuanced approach to policy-making with regard to the regulation of ENDS. ENDS use should be strongly discouraged among those who do not smoke cigarettes, especially young people. This can be achieved with a combination of strong marketing restrictions, flavour bans or limitations and taxation that targets products that the youth and people who do not smoke are likely to use. Considering that on-rampers took up ENDS young, the government might consider increasing the legal ENDS smoking age. Considering the large share of continuing dual consumers and failed off-rampers, the government should consider providing targeted cessation programmes to support people who vape and are trying to quit cigarettes.

## Data Availability

Data are available in a public, open access repository.
